# Oestrogen-selective modulation of FSH and LH secretion by pituitary gland

**DOI:** 10.1038/sj.bjc.6602292

**Published:** 2004-12-07

**Authors:** M F Mitwally, R F Casper, M P Diamond

**Affiliations:** 1 Division of Reproductive Endocrinology & Infertility, Department of Obstetrics & Gynecology, Wayne State University, Detroit, MI, USA; 2 Reproductive Sciences Division, Department of Obstetrics & Gynecology, University of Toronto, Toronto, Ontario, Canada

**Sir**,

We read the interesting article by Forward *et al* (2004) on the clinical and endocrine data for goserelin plus anastrozole in premenopausal women with advanced breast cancer. The authors presented interesting data regarding serum gonadotropin (FSH and LH) levels and androgen (testosterone, DHEAS and androstenedione) levels in 13 patients treated with the GnRH agonist, goserelin, plus the selective oestrogen receptor modulator, tamoxifen, followed by goserelin plus the aromatase inhibitor, anastrozole. The authors found that goserelin and tamoxifen led to a 90% fall in FSH levels down to 1.0 IU/L at 6 months (*P*<0.001). Once the therapy was changed to goserelin and anastrozole, there was a significant rise in the mean FSH level to 7.8 IU/l at 12 months (*P*<0.0001). Introduction of goserelin and tamoxifen resulted in an 89% reduction in mean LH levels (pretreatment *vs* 6 months=19.9 *vs* 0.3 IU/l; *P*=0.01), but with substitution of tamoxifen by anastrozole, no significant change in LH levels occurred.

With regard to androgen levels, the authors found a significant fall in testosterone and androstenedione, but not DHEAS, levels in association with substitution of tamoxifen by anastrozole.

In their explanation for these observations, the authors suggested that the rise in FSH was due to release of a negative feedback as a result of further reduction in oestradiol levels upon the introduction of an aromatase inhibitor, or a rebound phenomenon from coming off tamoxifen. Regarding the significant decline in androgen levels (testosterone and androstenedione), contrary to what is expected (a rise due to potential substrate accumulation secondary to inhibition of the aromatase enzyme), the authors provided little in the way of discussion.

We would like to propose a possible mechanism for the endocrine changes found in this study. Regarding the differential changes in gonadotropins (rise in FSH levels while LH levels remained suppressed) with substitution of tamoxifen by the aromatase inhibitor, anastrozole, we believe that involvement of the activin/inhibin/follistatin system could explain these changes.

Accumulating evidence suggests that oestrogen can modulate gonadotropin production through a direct effect on pituitary cells, independent of GnRH. This effect is thought to be mediated through the activin/inhibin/follistatin system (DePaolo, 1997; Nett *et al*, 2002; Welt *et al*, 2002; McNeilly *et al*, 2003). Activins are produced by a wide variety of tissues including the pituitary gland, and are specifically by gonadotropes (Roberts *et al*, 1989). Activins stimulate synthesis of FSH by a direct action on the gonadotropes (Mason *et al*, 1989). Follistatin, also produced by the pituitary gland, is an activin-binding protein and may decrease FSH synthesis by sequestering activin (Mather *et al*, 1993). Oestrogen was found to selectively suppress FSH production but not LH through an increase in follistatin/inhibin and a decrease in activin, independent of GnRH (Shupnik and Rosenzweig, 1991; DiGregorio and Nett, 1995; Baratta *et al*, 2001), mainly through activin suppression (Baratta *et al*, 2001).

Testosterone has been found to have the opposite effect of oestrogen on pituitary FSH (but not LH) production, that is, an increase in FSH by the gonadotropes. This effect was found to be mediated through locally produced activins (Burger *et al*, 2004). Potentially, testosterone may accumulate at the tissue level as a result of aromatase inhibition even in the absence of measurable changes in the serum. Such locally accumulated testosterone could exert a stimulatory effect on FSH production by the anterior pituitary through an activin-mediated effect. The reason why a more suppressive effect on FSH production occurred with the antioestrogen tamoxifen is probably related to its selective oestrogen receptor modulation effect, known to exert antioestrogenic effects at the level of the hypothalamus, but oestrogenic effects at the level of the pituitary gland (Gonzalez *et al*, 2000).

The significant decline in testosterone and androstenedione, but not DHEAS, in the face of aromatase inhibition is much harder to rationalise. It is possible that inhibition of oestrogen locally in the hypothalamus and pituitary by the aromatase inhibitor, together with GnRH receptor downregulation by goserelin, resulted in profound combined suppression of LH levels, especially at the level of bioactivity, which is not totally reflected in the immunoassay results.

It is important to mention that the evidence presented above came mainly from animal studies. However, if we can extrapolate from the available animal data, it is pertinent here to point out another potential mechanism for aromatase inhibitors to enhance the efficacy of controlled ovarian hyperstimulation (COH) protocols, as we have recently reported (Mitwally and Casper, 2002, 2003, 2004). The ability of the aromatase inhibitors to increase endogenous FSH production by the anterior pituitary, even with pituitary GnRH receptor downregulation (as in the present publication by Forward *et al*), is an exciting approach to improve the outcome of ovarian stimulation particularly in poor responders in *in vitro* fertilisation protocols. We believe this area of aromatase inhibitor use warrants further investigation.
